# Refractory Electrical Storm in the Absence of Structural Ischemic Heart Disease

**DOI:** 10.7759/cureus.6888

**Published:** 2020-02-05

**Authors:** Kashmala Khan, Francis Dimtri, Carlos Vargas, Christel Cuevas, Thomas Alexander

**Affiliations:** 1 Internal Medicine, Corpus Christi Medical Center, Corpus Christi, USA; 2 Cardiology, Corpus Christi Medical Center, Corpus Christi, USA

**Keywords:** electrical storm, ventricular fibrillation, vantricular tachycardia, monomorphic tachycardia, polymorphic tachycardia, life threatening arrhythmia, catheter ablation

## Abstract

Ventricular tachycardia (VT) is characterized as a ventricular rhythm with a QRS >120 milliseconds (ms) and >100 beats-per-minute (BPM) in the absence of an aberrant conduction. It is classified as sustained when lasting >30 seconds. Risk factors associated with the development of VT include increasing age and coronary artery disease with concurrent left ventricular dysfunction, other forms of structural heart disease and acquired or congenital abnormalities in the cardiac sodium, potassium or calcium channels. Diagnosing VT is challenging based on history and physical exam alone. Combination of electrocardiogram (EKG), electrolytes and cardiac enzymes, echocardiogram, cardiac catheterization, and electrophysiology testing are required to appropriately diagnose and characterize the etiology. The case below describes an 84-year-old female with a known history of symptomatic bradycardia status post pacemaker who presented to the emergency department (ED) after a routine device check which revealed VT with associated dyspnea. The patient did not do well with medical therapy and required ablative therapy to resolve VT.

## Introduction

An electrical storm refers to a life-threatening syndrome that entails a state of cardiac electrical instability with three or more episodes of ventricular tachycardia (VT) or ventricular fibrillation (VF) in a short period of time, typically within 24 hours [1]. Studies have shown a 7.4 fold increase in mortality in patients with electrical storm than other arrhythmias, with the mortality being the highest within the first three months of presentation [1]. Most patients with VT storm have underlying structural heart disease and it has been reported less frequently in patients with structurally normal hearts. Reversible triggers include drug toxicity, prolonged QT, electrolyte imbalances, acute myocardial infarction (MI), new or worsened heart failure and thyrotoxicosis [2]. Other factors include advanced age, male sex, low left ventricular ejection fraction, and New York Heart Association (NYHA) class II and III heart failure. The clinical presentation varies and ranges from cardiac arrest to vague symptoms of lightheadedness, palpitations and syncope. Patients are either seen with VT comprising of monomorphic tachycardia, polymorphic tachycardia or ventricular fibrillation. In an electrical storm, VT is more prevalent, seen in about 52% of the cases. VF contributes to 48% of cases seen [3].

## Case presentation

**Figure 1:**
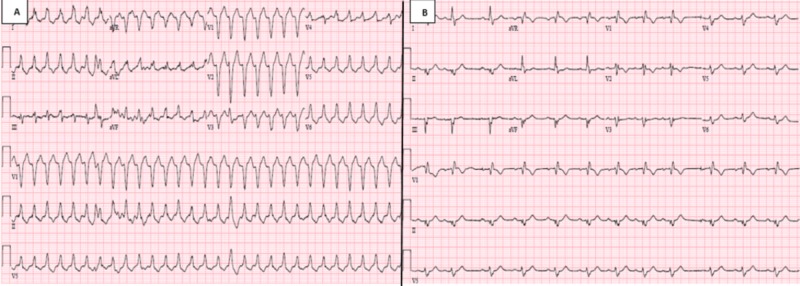
A: Electrocardiogram on presentation showing monomorphic wide complex tachycardia. B: Electrocardiogram after cardioversion, patient was briefly in sinus rhythm

This is an 84-year-old female with a past medical history significant for hypertension, type 2 diabetes mellitus (DM), peripheral neuropathy secondary to DM, hyperlipidemia, and hypothyroidism. Pertinent cardiac history includes bradycardia with syncope, status post dual chamber pacemaker placement eight months prior to presentation. During a routine cardiology follow up, she had a device check which confirmed VT with a rate of 180 beats per minute. Subsequently, paramedics were called and she was taken to the emergency department (ED). Her only complaints were shortness of breath and dizziness. She denied chest pain, palpitations and syncope. Initial electrocardiogram (EKG) showed wide complex monomorphic tachycardia (Figure 1A). She was given intravenous (IV) diltiazem, and an additional three doses of IV adenosine 12 mg in the ED by the ED physician with no response. She then received synchronized cardioversion at 200 joules, and was only briefly in sinus rhythm (Figure 1B).

She was subsequently treated with IV amiodarone bolus twice, followed by an amiodarone drip. She went back into VT, and was given IV lidocaine and cardioverted at 300 joules. An esmolol drip was started and she was transferred to the intensive care unit. Her initial labs showed a complete blood count with normal white blood count, no evidence of anemia, basic metabolic profile showed no electrolyte abnormalities, and normal thyroid stimulating hormone (TSH) levels were seen.

**Figure 2:**
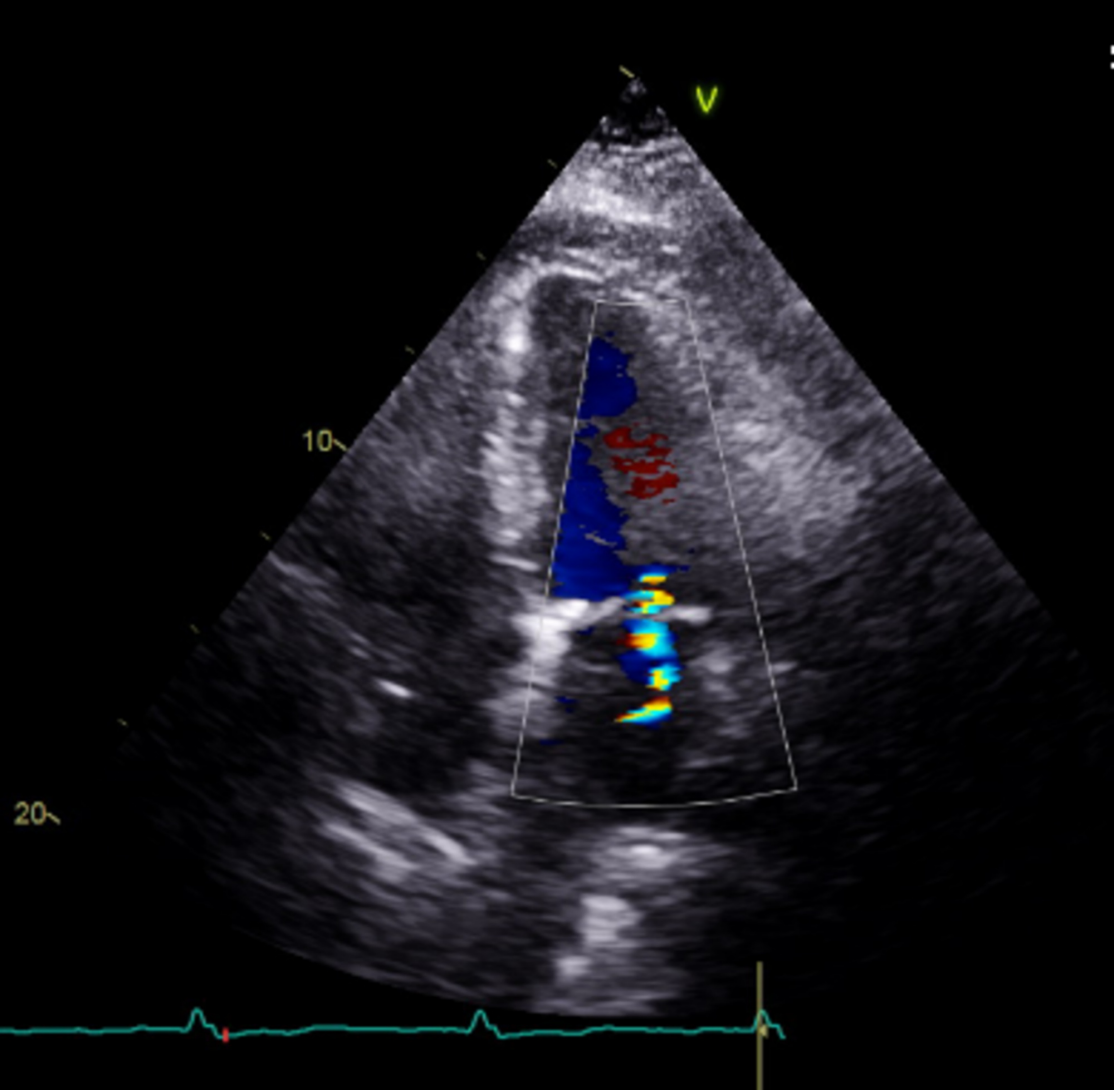
An echocardiogram showing an apical four chamber view There is normal appearance of the myocrdium, no evidence of infiltrative diseases, ischemic changes or scars. There is only mild mitral regurgitstion shown in the image.

Transthoracic echocardiogram was done to evaluate possible myocardial scar, left ventricular hypertrophy, infiltrative or dilated cardiomyopathies. The echocardiogram showed normal ventricular systolic function with an ejection fraction of 60%-65%. She did appear to have a diastolic filling pattern showing impaired relaxation, mild tricuspid and mitral regurgitation, and moderate pulmonary hypertension (right ventricular systolic pressure of 50.06 mmHg). There was no ventricular or septal thickening, no speckled appearance of the myocardium, or focal areas of edema suggesting an infiltrative disease process (Figure 2).

Myocardial perfusion imaging was performed which revealed homogenous radiotracer uptake on stress imaging, as well as no evidence of scar or ischemia and demonstrated normal ejection fraction. A left heart catheterization was performed to rule out ischemic cause, which revealed non-obstructive coronary artery disease. When the esmolol drip was stopped, breakthrough VT occurred. An electrophysiologist was consulted for possible ablation. 

**Figure 3:**
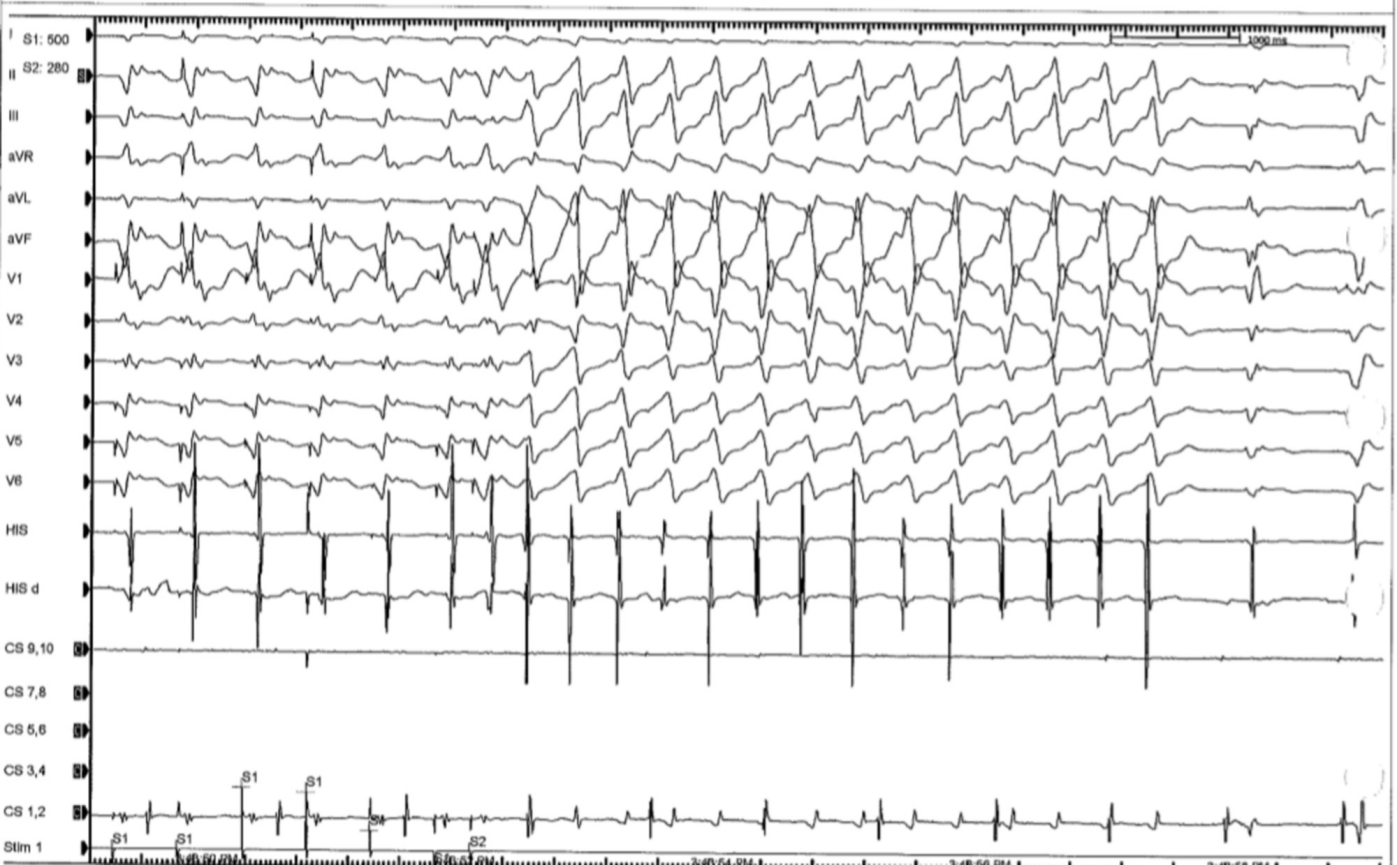
Pre-procedure electrocardiogram showing wide complex non sustained ventricular tachycardia

**Figure 4:**
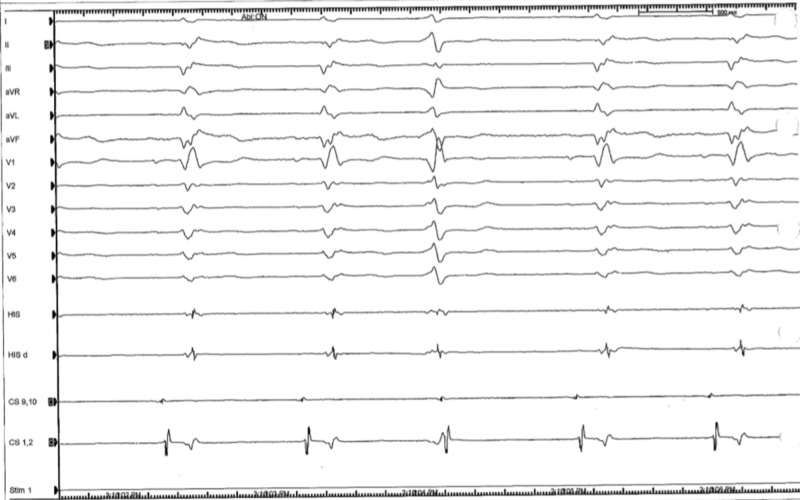
Intra-procedural electrocardiogram localizing the focus of arrhythmia, showing premature ventricular contractions

**Figure 5:**
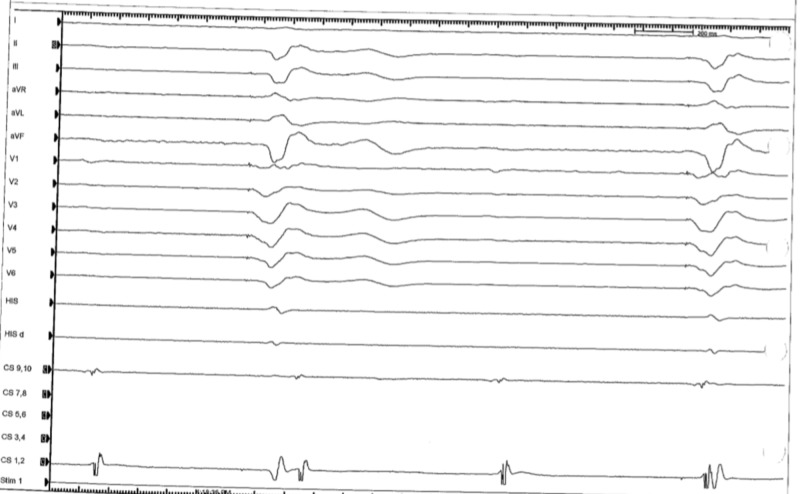
Intra-procedural electrocardiogram localizing focus of arrhythmia at the bundle of His and atriventricular node

Prior to the procedure, she was found to have a wide complex non-sustained ventricular tachycardia (Figure 3). During the procedure, the patient had frequent premature ventricular contractions (PVC) (Figure 4). The focus of the arrhythmia was localized at the bundle of His and the atrioventricular node (Figure 5).

**Figure 6:**
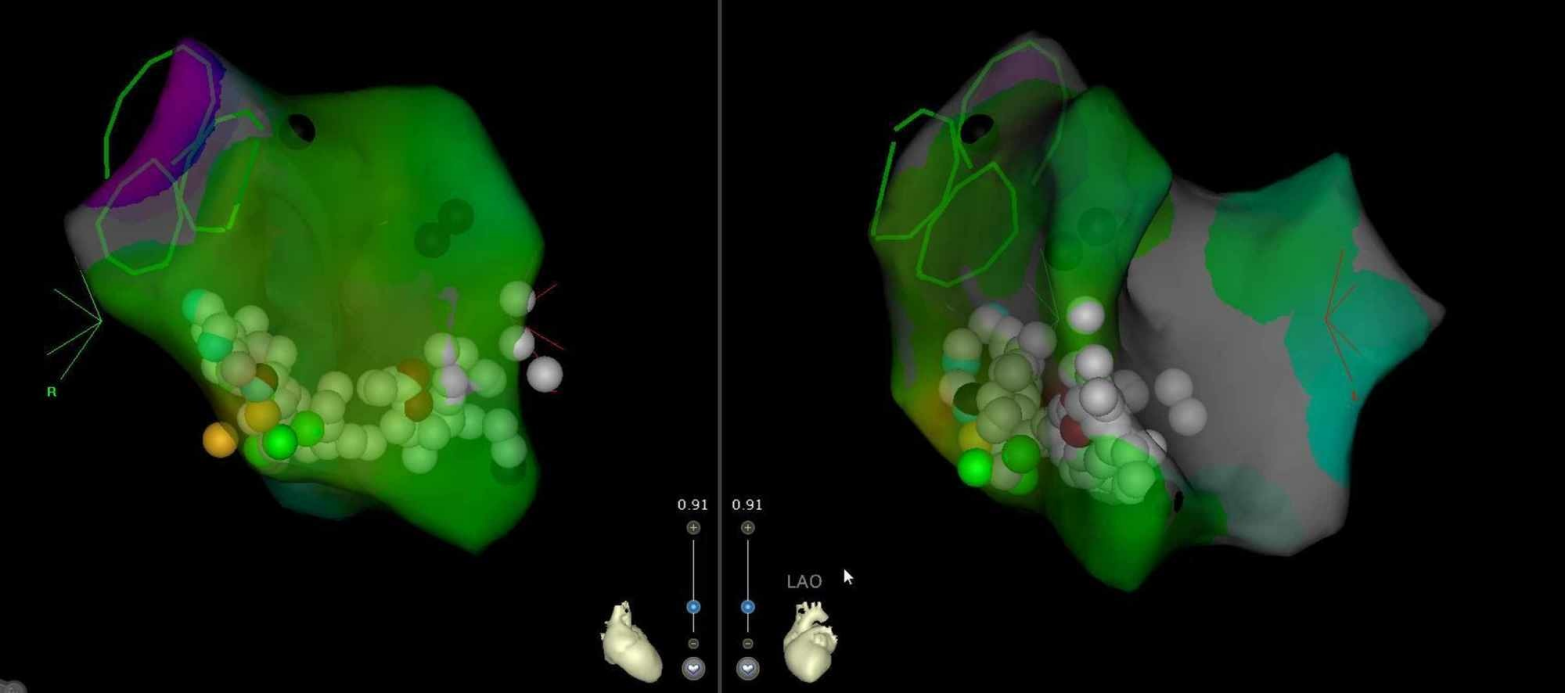
Intra-procedural 3D mapping showing activation around left and right parahisian area

Three-dimensional mapping was done that showed the site of earliest activation to be around the parahisian area (Figure 6). 

**Figure 7:**
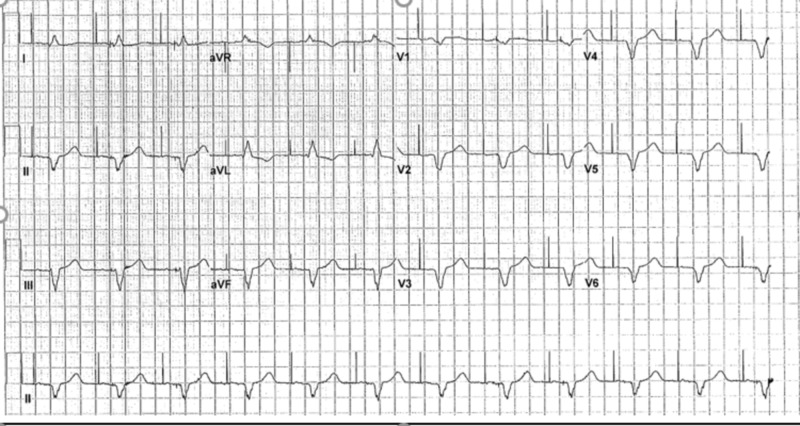
Post procedure electrocardiogram. Patient is pacemaker dependent

Aggressive ablation had to be performed which resulted in successful ablation of the VT but she became pacemaker dependent (Figure 7).

However, the patient remained stable and was discharged home the following day. During her post-hospitalization follow up at the cardiology outpatient clinic, she remained asymptomatic with no evidence of VT recurrence.

## Discussion

Electrical storm can present as either polymorphic tachycardia, monomorphic tachycardia, or ventricular fibrillation. This phenomenon is encountered more frequently in patients with structural heart disease and infiltrative diseases (eq. amyloidosis, sarcoidosis). Reversible causes include myocardial ischemia, electrolyte imbalances, thyrotoxicity, QT prolongation, and drug toxicity. It is also seen in reperfusion after acute ischemic episodes and myocardial scars that become potential arrhythmogenic foci. Polymorphic VT is usually seen with acute ischemia following reperfusion, hypertrophic cardiomyopathy and electrolyte imbalances [4]. Monomorphic VT is seen commonly where a myocardial scar acts like an arrhythmogenic focus [1]. The first step in management is to identify the initial rhythm and hemodynamic status of the patient. Unstable patients may require cardioversion and defibrillation [3]. The preferred pharmacologic treatment of monomorphic VT is the combination of amiodarone and a beta blocker. VT can be mistaken for supraventricular tachycardia with aberrancy, especially if the patient is hemodynamically stable with minimal symptoms [1]. This seemed to be the case in our patient as well. It is important to know the common causes of VT storm and try to investigate them while simultaneously treating the patient at initial presentation.

There is a subset of patients, like the patient is this case report, that can have a milder presentation despite having this potentially fatal arrhythmia. It is essential to investigate potential causes and treating them for arrhythmic cessation. However, if an arrhythmia is refractory to medical management, radiofrequency catheter ablation is the next step. Close outpatient follow up is recommended also due to the mortality risk that is greatest within the first three months after the procedure [1]. VT ablation has shown to successfully terminate VT storm. It is relevant to note that monomorphic VT is the most common manifestation of an electrical storm, followed by VF alone, mixed VT and VF and polymorphic VT. An electrical storm itself is a bad prognostic factor, and patients usually have high hospitalization rates [5]. Therefore, the initial rhythm must be well documented. As most patient receive antiarrhythmic therapy prior to catheter ablation, their effect can potentially continue during the procedure which could result in a less thorough ablation [6]. Antiarrhythmic therapy has been widely used for the treatment of VT storm. Patients with VT storm have ultimate cessation of the arrhythmia, reduced recurrence and survival benefit with catheter ablation [5]. The question rises whether initial antiarrhythmic should remain an elemental aspect of management or whether these patients should be referred for immediate catheter ablation. There is insufficient data and lack of randomized controlled clinical trials to determine if immediate catheter ablation without initial trial of pharmacologic therapy would be more beneficial.

## Conclusions

Here we have presented the case of a patient who had a very mild presentation in the setting of a life-threatening electrical storm. We have highlighted the different types of arrhythmias comprising electrical storm, and the factors most commonly leading to it. A step-wise approach is necessary in the management of an electrical storm; this includes establishing hemodynamic stability and recognizing the initial rhythm and reversible causes. It is important to differentiate wide complex ventricular tachycardia from supraventricular tachycardia with aberrancy. Most patients benefit from antiarrhythmic medication and catheter ablation. 

## Appendices

Disclaimer: This research was supported (in whole or in part) by HCA Healthcare and/or an HCA Healthcare affiliated entity. The views expressed in this publication represent those of the author(s) and do not necessarily represent the official views of HCA Healthcare or any of its affiliated entities.
